# The Minimal Autoinhibited Unit of the Guanine Nucleotide Exchange Factor Intersectin

**DOI:** 10.1371/journal.pone.0011291

**Published:** 2010-06-24

**Authors:** K. Farid Ahmad, Wendell A. Lim

**Affiliations:** Department of Cellular and Molecular Pharmacology, University of California San Francisco, San Francisco, California, United States of America; University of Queensland, Australia

## Abstract

Intersectin-1L is a member of the Dbl homology (DH) domain guanine nucleotide exchange factors (GEF) which control Rho-family GTPase signaling. Intersectin-1L is a GEF that is specific for Cdc42. It plays an important role in endocytosis, and is regulated by several partners including the actin regulator N-WASP. Intact intersectin-1L shows low Cdc42 exchange activity, although the isolated catalytic DH domain shows high activity. This finding suggests that the molecule is autoinhibited. To investigate the mechanism of autoinhibition we have constructed a series of domain deletions. We find that the five SH3 domains of intersectin are important for autoinhibition, with the fifth domain (SH3(E)) being sufficient for the bulk of the autoinhibitory effect. This SH3 domain appears to primarily interact with the DH domain. We have determined the crystal structure of the SH3(E)-DH domain construct, which shows a domain swapped arrangement in which the SH3 from one monomer interacts with the DH domain of the other monomer. Analytical ultracentrifugation and gel filtration, however, show that under biochemical concentrations, the construct is fully monomeric. Thus we propose that the actual autoinhibited structure contains the related intramolecular SH3(E)-DH interaction. We propose a model in which this intramolecular interaction may block or distort the GTPase binding region of the DH domain.

## Introduction

Rho family GTPases are the master regulators of the actin cytoskeleton. Consequently, they coordinate diverse cellular processes including motility, adhesion, cytokinesis, phagocytosis, and neurite extension and retraction [1] [2] [3] [4] [5]. These proteins function as switches that adopt different conformations in the GDP- and GTP-bound states [6] [7] [8]. However, only the GTP-bound state can interact with downstream effectors and transduce signal [4] [9]. Enzymes known as guanine nucleotide-exchange factors (GEFs) catalyze the exchange of bound GDP for GTP, and thus convert GTPases to their active state. GEFs thus link diverse upstream input signals to control the actin cytoskeleton.

Like many other modular signaling proteins, most GEFs contain a conserved catalytic domain (which acts on GTPases) embedded within a more complex set of other domains that act to regulate or localize this catalytic domain. The largest family of GEF proteins contain a catalytic Dbl-homology (DH) domain [1]. These proteins are characterized by a conserved region of 300 amino acids, consisting of a ∼200 residue Dbl homology (DH) domain followed by a ∼100 residue pleckstrin homology (PH) domain [10]. The DH domain is both necessary and sufficient for the nucleotide exchange activity of Dbl family proteins [11] [12] [13] [14]. Structural studies have revealed that DH domains interact and reshape the conformationally variable “switch regions” of Rho GTPases, disrupting both magnesium and GDP binding [6] [7]. As a result of the relatively high intracellular concentrations of GTP (∼20 fold higher than GDP), GTP preferentially binds the nucleotide-free GTPases, leading to GTPase activation [15].

The function of the PH domain in regulating nucleotide exchange is less understood, but its invariant positioning immediately C-terminal to the catalytic DH domain suggests an important role. The PH domain is present in many intracellular signaling proteins, and has been shown to bind both proteins and phosphoinositides [16]. Earlier studies had suggested a role for the PH domain in membrane targeting [17]. However, PH domains of Rho GEFS bind phosphoinositides with low affinity and little specificity, and it has been demonstrated that other domains outside of the DH-PH cassette make a more substantial contribution to cellular distribution [18] [19] [20]. Instead, it is now thought that the PH domain plays a role positively or negatively modulating DH-domain nucleotide exchange activity. Several reports have shown that DH-PH fragments, both *in vivo* and *in vitro*, have greater nucleotide exchange activity than the respective DH domains alone [21] [22]. For some instances, this observation has been supported with structural data. Indeed, crystal structures have shown that the PH domains of Dbs and LARG make direct contacts with the bound GTPase [8] [23]. However, the crystal structures of Tiam-Rac1 and Intersectin-Cdc42 show no direct interactions between the PH domain and DH-bound GTPase, suggesting that this mechanism of activation is not universal [6] [7]. There are also several examples in which the PH domain has an inhibitory effect on DH domain mediated nucleotide exchange [24] [25] [26]. For instance, structural studies reveal the Sos PH domain makes a direct interaction with the GTPase binding region of the DH domain, hindering GTPase binding [27].

Outside of the DH-PH domain cassette, Rho GEFs are highly diverse in sequence. Rho GEFs are generally large proteins (>1000 amino acids), and often contain several domains involved in their localization, association with other proteins, and regulation of nucleotide exchange activity [1]. Many Rho GEFS are constitutively activated by the truncation of residues N-terminal to the DH domain (Vav1, Dbl, Asef) [13] [28] [29] or by the truncation of residues C-terminal to the PH domain (P-Rex, Lbc) [30] [31]. This finding implies that these regions function as negative, intramolecular (autoinhibitory) regulators of DH-domain function. Post-translational modifications, lipid, and protein-protein interactions have been shown to modulate GEF activity, presumably by disrupting such intramolecular interactions [1]. For example, structural studies have shown that the Vav1 DH domain forms a core autoinhibitory intramolelcular interaction with an Acidic region immediately N-terminal. Phosphorylation of a tyrosine in the Acidic region disrupts the intramolecular interaction and opens the DH domain to GTPases [13]. It has been recently shown that this core interaction is further strengthened by interactions between other Vav1 domains outside of the Acidic-domain/DH domain cassette, and that phosphorylation disrupts these modulatory contacts [32]. Although many Rho GEFs are thought to be regulated through intramolecular interactions, there are very few whose mechanism of autoinhibition and activation are understood at the level of Vav1.

**Figure 1:**
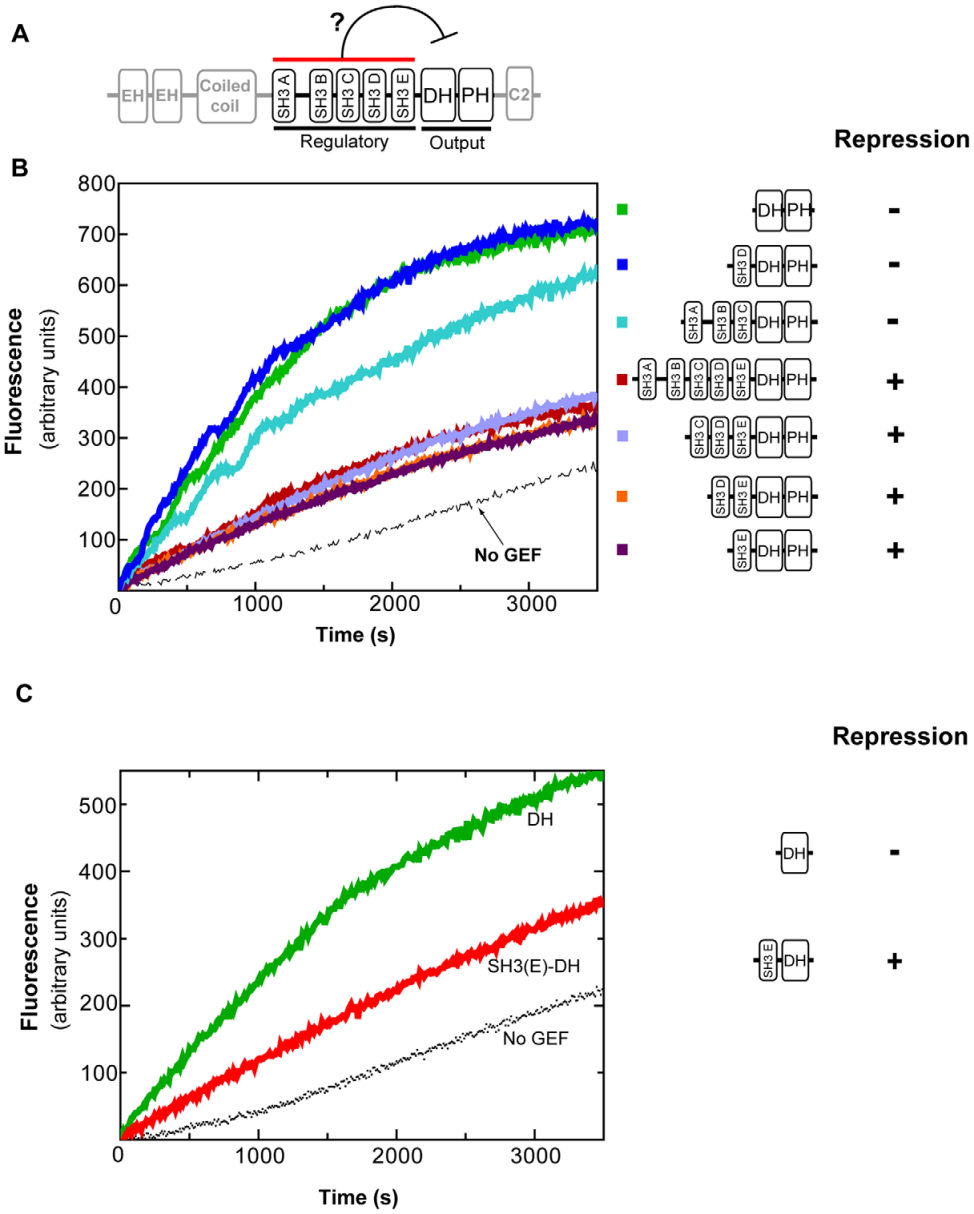
The minimal autoinhibited unit of intersectin-1L is comprised of the SH3(E)-DH domain fragment (1141–1435). a) Schematic of intersectin 1L domain structure, with regulatory (SH3 domains) and output domains (DH-PH) highlighted. b) *In vitro* fluorescence assay showing loading of mant-GDP onto GTPase Cdc42 by DH-PH domain-containing fragments of intersectin 1L. Fragments containing SH3(E) domain exhibit approximately 3-fold repression compared to the DH-PH domain. c) *In vitro* fluorescence assay comparing loading of mant-GDP onto GTPase Cdc42 by intersectin(SH3(E)-DH) versus intersectin(DH). SH3(E)-DH is repressed approximately 2.5-fold.

Intersectin-1L is a large (∼190 kDa), modular, endocytic scaffolding protein that also has GEF activity for Cdc42. It is composed of two N-terminal Eps15-homology (EH) domains, a putative coiled-coiled domain, five SH3 domains related with a sequence identity of 30%–40% (SH3 A, B, C, D, E), followed by the DH and PH domains and a carboxyl-terminal C2 domain (figure 1a) [31]. Intersectin-1L is unusual in that the DH and PH domains are not fundamental to its cellular role. It is a neuronal splice variant of the ubiquitously expressed intersectin-1S, a shorter protein lacking the DH, PH, and C2 domains [33] [34] [35]. Through the EH domains, intersectin-1 interacts with epsins and is thus targeted to clathrin-coated pits [33] [36]. Subsequently, dynamin and synaptojanin are recruited to these endocytic structures through an interaction with a subset of intersectin-1 SH3 domains [31] [37]. Overexpression of intersectins or its SH3 domains alone have been shown to inhibit clathrin-mediated endocytosis, presumably by sequestration of dynamin from endocytic complexes [34] [36] [38]. Based on these observations, it is proposed that intersectin-1 can act as a scaffolding protein that targets and holds proteins of the endocytic machinery at specialized zones of the plasma membrane.

A number of studies have addressed the pathways downstream of the intersectin-1L GEF activity, which is highly specific for the Rho GTPase Cdc42 [7]. Important downstream effectors for activated Cdc42 include the Wiskott-Aldrich syndrome protein (WASP) and its neuronal isoform, N-WASP. Activated WASP proteins bind to and activate the Arp2/3 protein complex, causing localized actin assembly and subsequent filopodia formation [39] [40] [41] [42]. Accordingly, it has been demonstrated that microinjection of the intersectin-1L DH domain activates Cdc42 and stimulates filopodia in cultured fibroblasts [43]. Emerging data has also suggested an important role for localized actin polymerization in vesicle endocytosis [37] [44] [45]. Specifically, it has been shown to generate the propulsive force driving transport of endocytic vesicles from the cell surface into the cytoplasm. The assembly of these actin comet tails depends on the activation of N-WASP [46].

As seen for many GEFS, the ability for intersectin-1L to mediate nucleotide exchange is inhibited in the full-length protein [43] [47]. The five SH3 domains found in intersectin-1L have been implicated in autoinhibition. Intriguingly, the N-WASP proline-rich region has been found to interact with the intersectin SH3 domains [47] [43]. Furthermore, the binding of the N-WASP proline-rich region to intersectin-1L stimulates the Cdc42 exchange activity of immunoprecipitated full-length protein [43].

However, activation by the proline-rich region of N-WASP has not been demonstrated with recombinant intersectin-1L fragments, suggesting that other, unidentified components present in cell extracts may be necessary for activation of DH domain nucleotide exchange activity *in vivo* [47]. Furthermore, mutation of the PxxP binding groove of the SH3E domain does not interfere with SH3 domain-mediated inhibition of Cdc42 nucleotide exchange *in vitro* [47]. Such information suggests that the PxxP-binding groove is not involved in inhibiting nucleotide exchange activity.

To understand the mechanism of intersectin regulation, we have used deletion analysis to map the minimal autoinhibited fragment of the protein. A crystal structure of this fragment has been determined, which gives insight into the molecular mechanism of autoinhibition and activation of this protein.

## Results

### Mapping the domains necessary for intersectin autoinhibition

The intersectin-1L SH3 domains negatively regulate the *in vitro* GEF activity of the adjacent DH domain [47]. However, it is not absolutely clear which SH3 domains participate in intramolecular interactions, which output domain they bind to (DH or PH or both), and which set of SH3 domains are sufficient for inhibition. Initially GST-DH, GST-PH, and GST-DHPH proteins were used as bait to screen binding, in trans, to both each SH3 domain individually and all five SH3 domains together. However, unlike what has been reported in a previous study [47], no interaction was observed. Although we utilized a slightly shorter amino termini for our SH3(ABCDE) fragment (starting at amino acid 741 versus 693) the authors of the previous study were also able to demonstrate binding of the individual SH3 domains. Our DH-PH domain fragment is slightly shorter at the amino termini (starting at amino acid 1222 versus 1214), which may account for the discrepancy. Surface plasmon resonance methods were also unable to measure an interaction in trans between the output and inhibitory domains. As well, fragments SH3(ABCDE) and SH3(E) were unable to repress the nucleotide exchange activity of the isolated DH-PH domain in trans, with concentrations of inhibitory protein up to 25 µM (data not shown). These observations suggest that the interaction between the SH3 domains and the DH-PH domain is relatively weak. Although it is routinely observed in pull-down assays that the intersectin DH domain binds with high affinity to nucleotide-free Cdc42 [47], the affinity of nucleotide-bound Cdc42 is much weaker [48] [49]. Interactions between the DH/DH-PH domain and SH3 domains most likely are only measurable when they are each in a high local concentration, such as when they are linked. Accordingly under these conditions, it is more likely that the SH3-DH domain interaction will be of sufficient affinity to repress the nucleotide exchange activity of the attached DH-PH domain on Cdc42. Therefore, to identify the domains involved in autoinhibitory interactions, DH-PH domain constructs containing the amino-terminal SH3 domains (A, B, C, D, and E) attached in cis were assayed for GEF activity *in vitro*. This activity was monitored by following the increase in relative fluorescence due to the loading of a fluorescent GDP into Cdc42. Relative to the isolated DHPH domain, the GEF activity of fragments SH3(ABCDE)-DH-PH, SH3(CDE)-DH-PH, SH3(DE)-DH-PH, and SH3(E)-DH-PH were repressed approximately 3 fold. This result demonstrates that the SH3(E) domain is both necessary and sufficient for DH-PH repression. In support of this observation, DH-PH constructs lacking the SH3(E) domain (SH3(ABC)-DH-PH, SH3(D)-DH-PH) were not significantly repressed. They exhibited GEF activity comparable to the DH-PH domain alone (figure 1b).

The PH domains of Rho GEFs Dbl and P-Rex have been shown to participate in autoinhibitory interactions with domains outside of the DH-PH cassette [28] [50]. Presumably, such intramolecular interactions occlude or distort the DH domain. To determine the role, if any, of the PH domain in intersectin-1L autoinhibition, the ability of the SH3(E) domain to repress DH domain function in the absence of the PH domain was tested. It is important to note that the activity of the isolated DH domain was consistently lower than that of constructs that also included the PH domain, an observation consistent with previous studies [47]. Nonetheless, GEF activity of SH3(E)-DH domain fragment was repressed approximately 2.5 fold compared to the isolated DH domain (figure 1c). This level of repression is comparable to the repression the DH-PH domain by SH3(E) in cis, demonstrating that the bulk of autoinhibition of the intersectin-1L GEF activity is mediated by an intramolecular interaction between the SH3(E) domain and the DH domain.

### The crystal structure of SH3E-DH fragment of intersectin-1L

**Figure 2:**
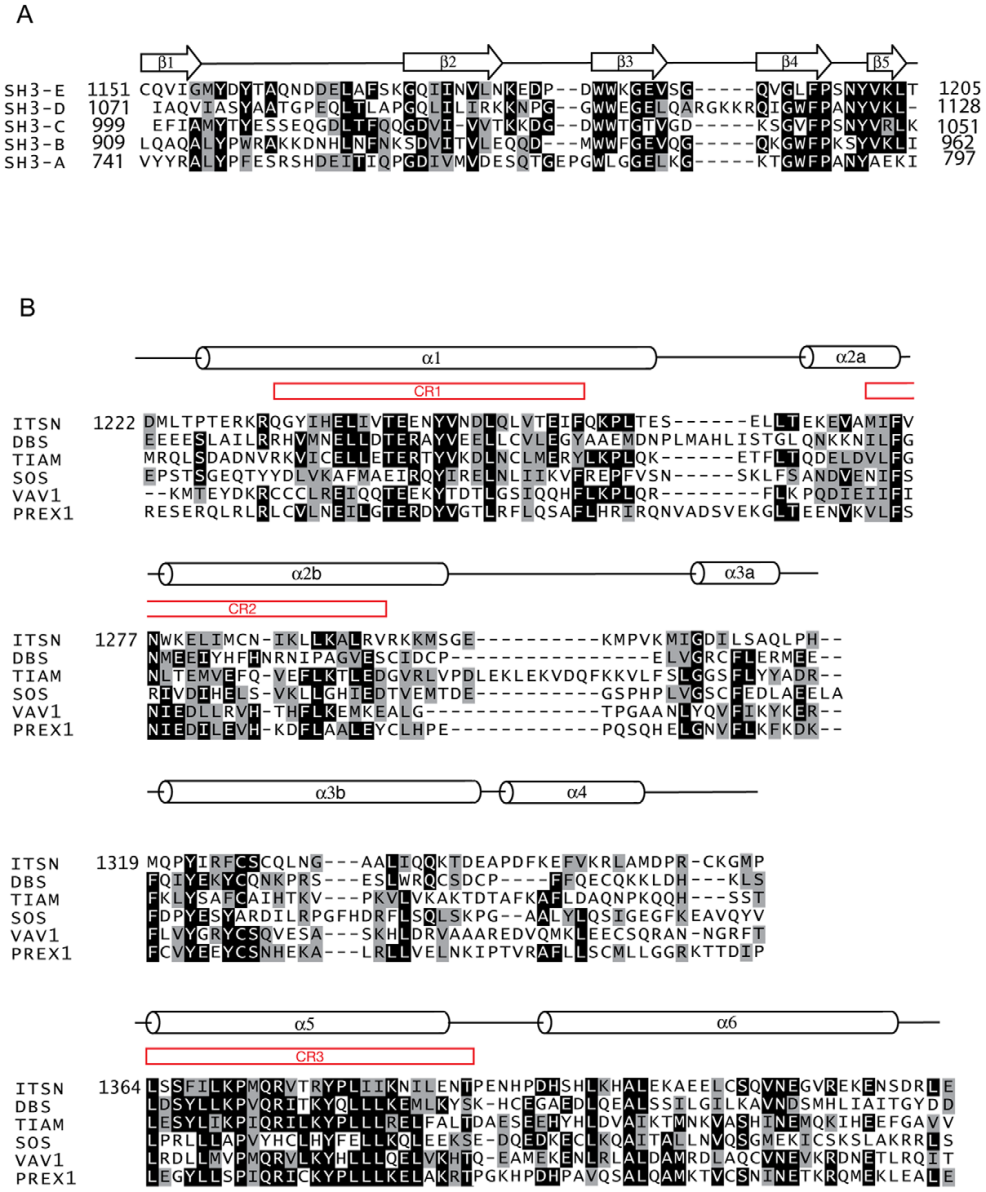
Observed secondary structure of intersectin-1L SH3(E)-DH (1141–1435). a) Sequence alignment of murine intersectin SH3 domains A through E. The observed secondary structure of SH3(E) is indicated above the sequence. The numbering is according to murine intersectin-1L. Black and gray backgrounds are used to indicate identical and/or conserved residues found in at least 50% of the proteins at a given position. b) Sequence alignment of selected DH domains and the observed secondary structure of the intersectin-1L DH domain. Residues from intersectin-1L are numbered. Highly conserved regions among all DH domains are labeled in red as CR1-3.

**Figure 3:**
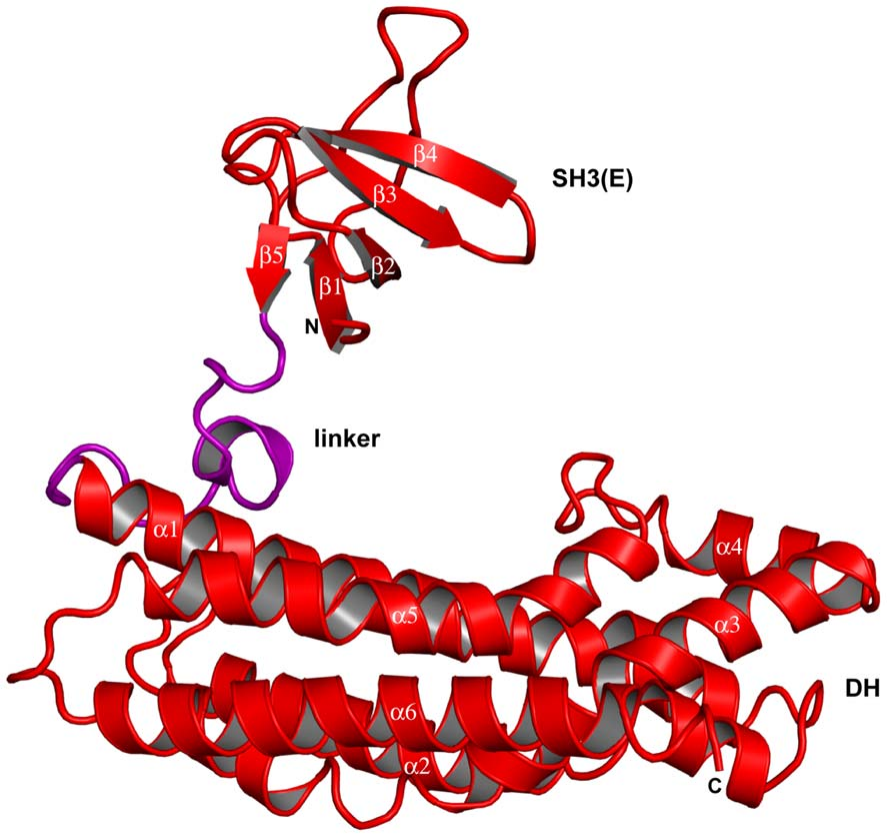
Ribbon diagram of the intersectin-1L SH3(E)-DH (1141–1435) monomer. The SH3(E) domain does not interact with the DH domain in the same chain. The linker joining the SH3(E) domain and the DH domain (1204–1225) is colored in purple. The secondary structure elements of the SH3(E) domain and the DH domain are indicated.

**Figure 4:**
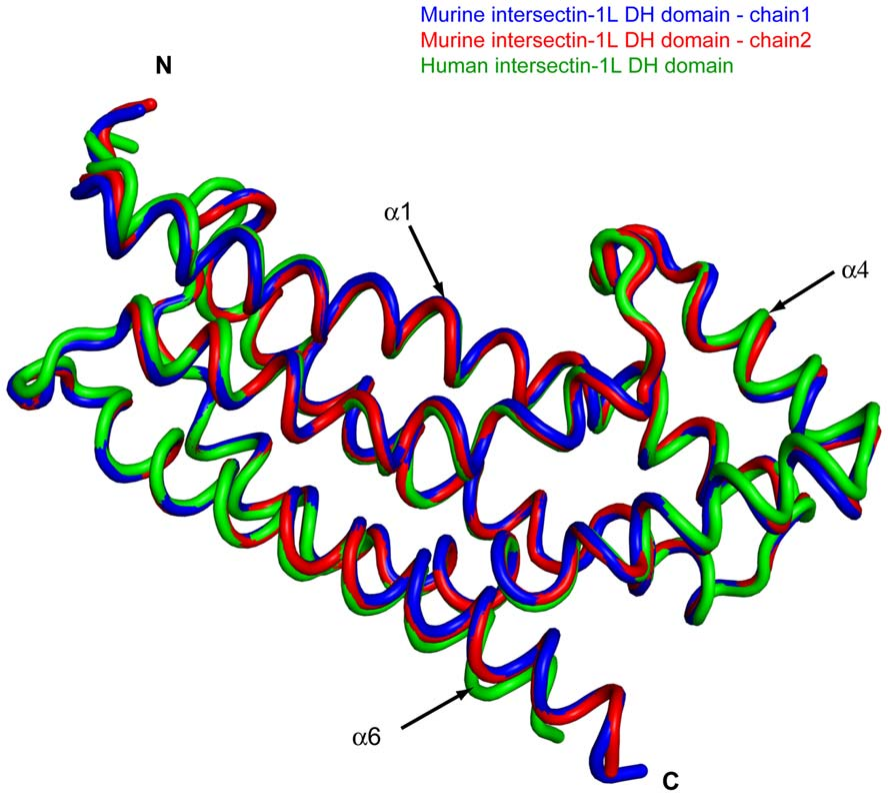
Superposition of intersectin-1L DH domains. The two “repressed” murine intersectin-1L DH domain monomers (blue, and red) and “active” human intersectin-1L DH domain (green). The least-squares superposition of these three chains result in an average pairwise rmsd value of less than 1.0 Å.

Both SH3(E)-DH-PH and SH3(E)-DH fragments of intersectin-1L were subjected to extensive crystallization trials. We were unable to crystallize the SH3(E)-DH-PH fragment, but the SH3(E)-DH fragment (1151–1431) yielded well-formed, hexagonal-shaped crystals, belonging to space group P3_2_21 with two chains in the asymmetric unit. Molecular replacement phasing was used to determine the three-dimensional structure to 2.4 Å (3JV3) ((table 1). The SH3(E) domain of intersectin-1L (1151–1203) adopts the typical fold of SH3 domains, with five anti-parallel β-strands packed to form two perpendicular β-sheets [51]. The SH3(E) domain in one of the two protein chains is disordered and thus was not modeled. The linker connecting the SH3(E) domain and DH domain (1204–1225) is well ordered in both chains of the asymmetric unit, with residues 1210–1214 unexpectedly forming a short α-helix. The DH-PH domain fragment utilized in Zamanian et al. commences at residue 1214 [47]. In their study, they were able to demonstrate binding of the intersectin SH3 domains to both the intersectin DH and the DH-PH domains in trans. Residues 1215–1221 are well ordered yet do not contribute to the established DH domain fold. Furthermore, they do not make any contacts with either the SH3(E) domain or the DH domain. Thus, it is unlikely that exclusion of residues 1214–1221 in our DH/DH-PH fragments explain the discrepancy between the two studies. The DH domain (1226–1431) is an elongated helical bundle structurally homologous to other DH domains of known structure. Composed of six major helical segments (α1–α6), there are three highly conserved regions in all DH domains (CRs1-3). CR1 (α1) and CR3 (α5) together with parts of α3 and α6 form the major binding surface for Cdc42 (figure 2b) [8]. Of note, the N-terminal portion of α5 is implicated in dictating GTPase specificity [7]. CR2 (α2) is on the opposite side of the helical bundle relative to CRs1 and 3 and is thought to stabilize the helical bundle (figure 3) [8]. The structure of the “repressed” DH domain as reported in this study is highly similar to the “active” DH domain observed in the crystal structure of human intersectin-1L complexed with Cdc42 [7]. A least squares superposition of the crystallographically unique DH domain fragments reported here with the DH domain of human intersectin-1L complexed with Cdc42 result in an average pairwise RMSD value of less than 1.0 Å. Furthermore, difference-distance matrix analysis does not reveal any local conformational changes between the different intersectin-1L DH domain structures (figure 4) [52].

**Table 1:** Crystallographic statistics for intersectin SH3(E)-DH domain structure.

Intersectin SH3(E)-DH domain	
**Data collection**	
Space group	P3_2_21
Cell dimensions (Å)	a = b = 67.044, c = 341.818, α = β = 90°, γ = 120°
Wavelength (Å)	1.11587
Resolution (Å)	2.4
Total reflections	274736
Unique reflections	36102
Redundancy	7.6
Completeness	99.5% (97.1%)
<I>/<σI>	38.7 (3.2)
R_sym_	5.0% (38.6%)
**Refinement**	
Resolution (Å)	50–2.4
Data cutoff (F/σF)	0
R_work_/R_free_ (%)	24.75/28.71
Number of protein atoms/waters	4062/78
Average B-factor	75.21
RMSD bond lengths (Å)	0.0096
RMSD bond angles	1.39°

Numbers in parentheses refer to the high resolution shell (2.49–2.40 Å ). R_sym_ = 100 x (∑_h_∑_I_|I_h,i_ –I_h_|)/∑_h_∑_i_I_h,i_ for the intensity I of i observations of reflection h. I_h_ is the mean intensity of the reflection. <I>/<σI> = mean intensity/mean standard deviation. R_work_ = 100 x ∑ |F_obs_ - F_calc_|/∑|F_obs_|, where F_obs_ and F_calc_ are the observed and calculated structure factor magnitudes, respectively. R_free_ is the same as the R_work_, but is calculated from 5% of the reflection data excluded from the refinement.

**Figure 5:**
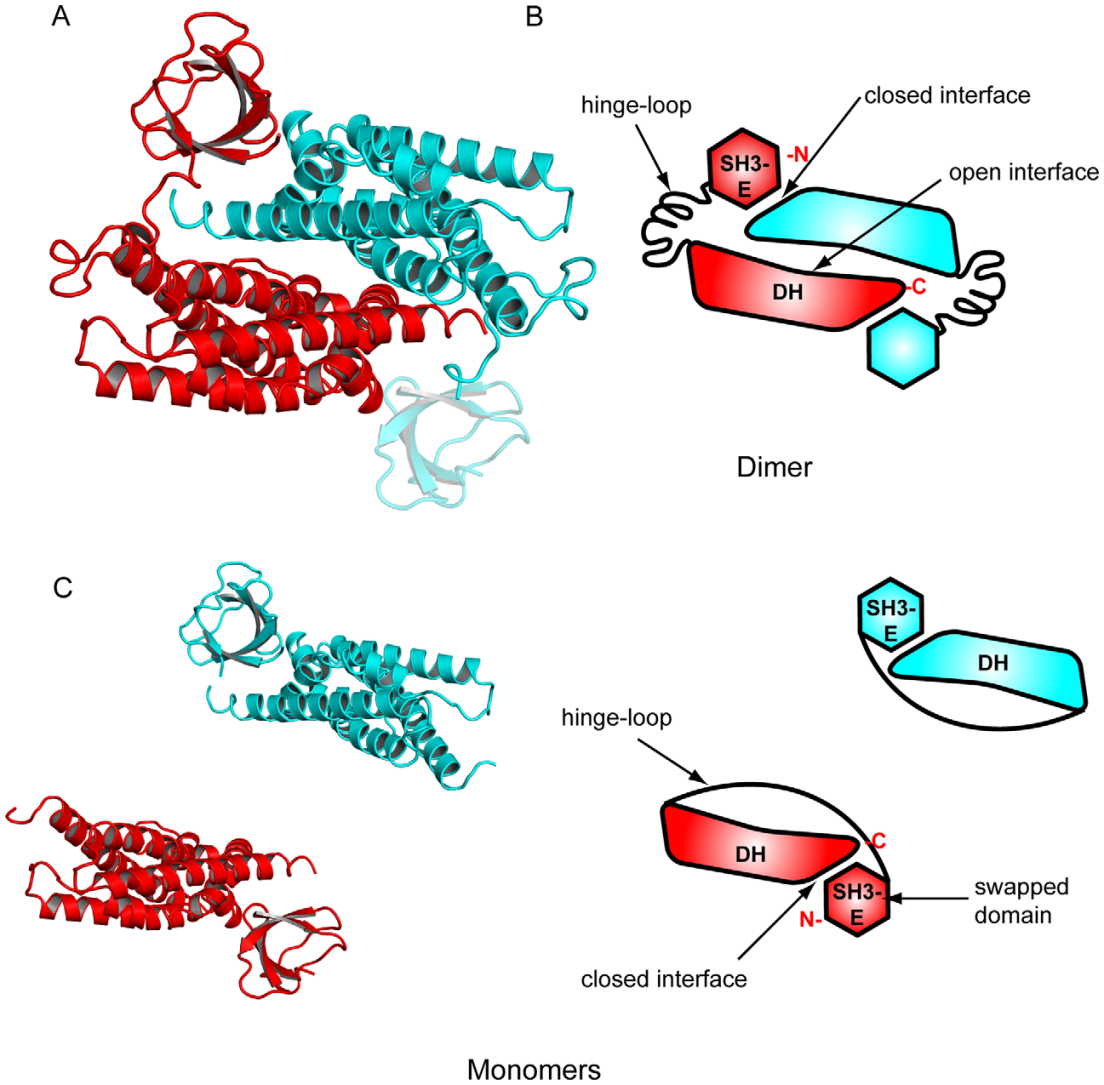
Structure of the intersectin-1L SH3(E)-DH domain homodimer. a) Ribbon diagram of the intersectin-1L SH3(E)-DH domain homodimer. One monomer is colored red, the other blue. The SH3(E) domain of the blue monomer was not present in electron density maps and is modeled here in a transparent blue. b) Schematic diagram of the intersectin-1L SH3(E)-DH domain homodimer, illustrating the terms related to 3D domain swapping. For simplicity, only elements pertaining to one chain are labeled. The closed interface is the interface between the SH3(E) domain and the DH domain. It exists in both the monomer and the domain-swapped dimer. The open interface is the interface between DH domain monomers. It exists only in the domain-swapped dimer, but not in the monomer. The hinge loop connects the SH3(E) domain and the DH domain. It adopts different conformations in the monomer and the domain-swapped dimer. c) Schematic diagram of possible domain organization in SH3E-DH domain monomer. The SH3(E) domain (the swapped domain) forms an intramolecular interaction the DH, and in a manner identical to that of the SH3(E)-DH domain intermolecular interaction in the dimer.

Interestingly, the SH3(E) domain from one chain interacts with the DH domain of the other chain, an arrangement suggestive of a domain-swapped dimer (figure 5). Domain swapping is a mechanism for two monomers to form a dimer by exchanging an identical structural element. Using the terminology used for 3D domain-swapped proteins [21] the intersubunit contacts can be grouped into two classes: a pair of domain-swapped “closed interfaces” involving interactions between the SH3(E) domain and the DH domain and a central “open interface” involving interactions between the DH domain monomers. The closed interface exists in both the monomer and domain-swapped oligomer, while the open interface exists only in the domain-swapped dimer. Thus in the case of the putative SH3(E)-DH domain monomer, the SH3(E) domain would interact with the DH domain of its own chain, in a manner essentially identical to that of the SH3(E)-DH domain intermolecular interaction in the dimer. The “hinge-loop” segment that links the swapped and main domain (amino acids 1204–1225) would adopt different conformations in the monomer and the domain-swapped dimer.

### DH-DH interface (open interface)

**Figure 6:**
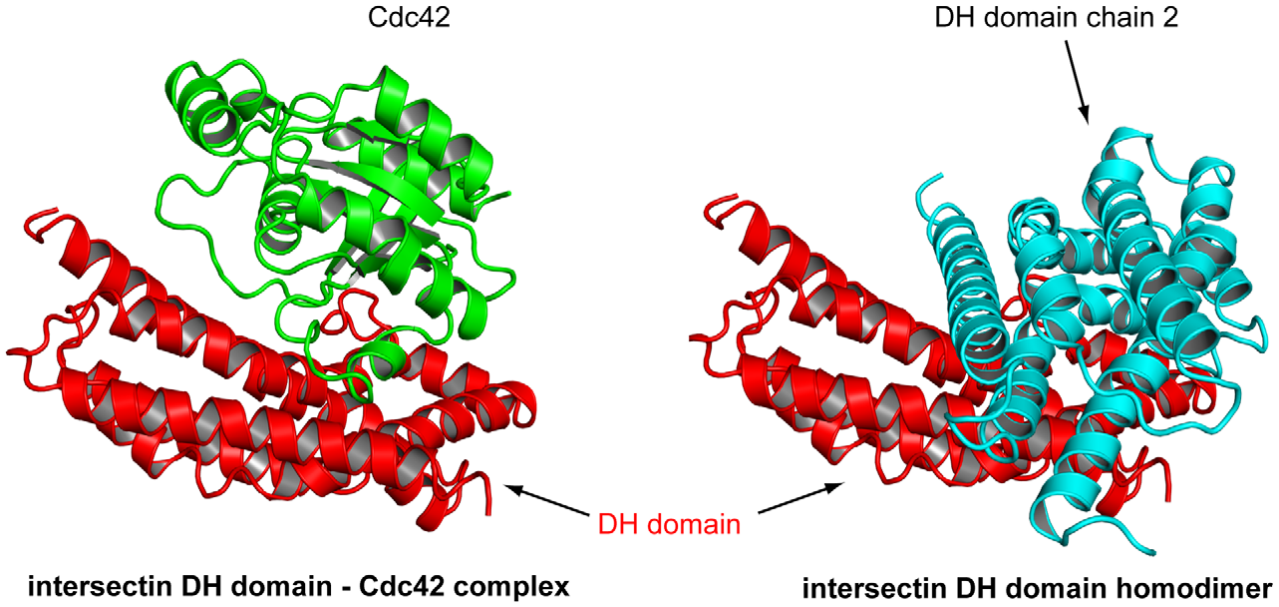
Parallel display of the intersectin-1L DH domain homodimer and intersectin-1L DH domain-Cdc42 complex. The DH domain in red is in the same orientation in both structures. The DH domain of the second monomer of the intersectin DH domain homodimer is in blue. The Cdc42 molecule in the DH domain-Cdc42 complex is green. The binding site for Cdc42 on the DH domain is occluded by DH-DH homodimer formation.

Approximately 10% of the DH monomer surface area (1040 Å^2^) is buried upon dimerization between DH domain monomers and involves mostly contacts between polar and charged residues. The hydrophilic nature of the dimerization interface is consistent with that found in proteins that readily exchange binding partners. The interaction surface of the DH domain monomers has contributions from the central portion of α1 (CR1), the c-terminal portion of α3, α5 (CR3), and one face of the c-terminus of α6. This surface overlaps highly with the Cdc42 binding surface (figure 6) [7]. Cdc42 has two regions that have different conformations in the triphosphate-bound, compared to the diphosphate-bound, state. These “switch” regions interact extensively with regions in the intersectin-1L DH domain that would be inaccessible upon DH-domain dimer formation. In particular, switch 1 of Cdc42 interacts with CR1 and CR3 of intersectin-1L. A highly conserved glutamate in CR1 (residue 1237 in murine intersectin-1L), shown to be crucial for Cdc42-intersectin-1L complex formation and nucleotide-exchange formation, is buried upon DH dimer formation. Switch 2 predominantly contacts CR3 and portions of α6, both of which make significant DH-DH domain dimer interactions [7]. The isolated intersectin-1L DH domain is exclusively monomeric in solution (data not shown). Other DH domains have been shown to dimerize (notably Dbl, Dbs, and Tiam) [6] [8] [53]. However, unlike the intersectin-1L DH domain, the dimerization interface is composed of residues from CR2 and is on the opposite side of the molecule relative to the GTPase binding site.

**Figure 7:**
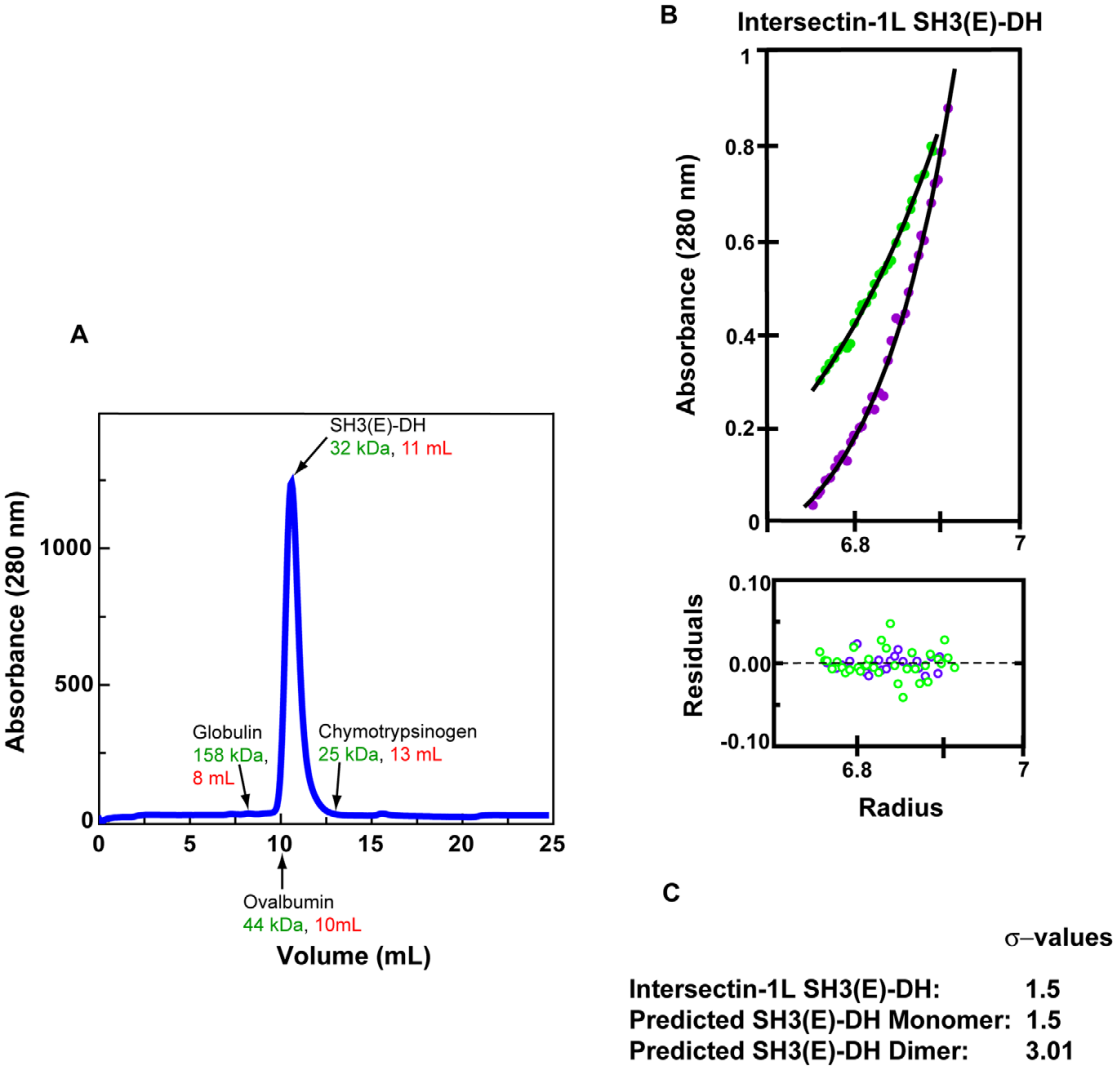
Intersectin(SH3(E)-DH) is monomeric. a) Elution profile of Intersectin(SH3(E)-DH) from a Superdex-75 size-exclusion column. Intersectin(SH3(E)-DH) elutes as a single peak between the elution volumes of ovalbumin (44 kDa) and chymotrypsinogen (25 kDa). b) Analytical ultracentrifugation (AUC) analysis of intersectin(SH3(E)-DH). The top curve is at 14,000 rpm and the bottom at 20,000 rpm. Shown below are the residuals for fits to the data. The data fit to an effective molecular weight (σ) of 1.5. c) Predicted σ values of different oligomeric states of intersectin(SH3(E)-DH). A σ value of 1.5 correlates to a molecular weight of 32 kDa, based on AUC analysis of the 12.9 kDa protein profillin (σ = 0.6092).

As a result of the high degree of overlap between the observed DH dimerization interface and Cdc42 binding surface, SH3-domain induced dimerization of the intersectin-1L DH domain possibly could lead to DH-domain GEF inhibition. However, both analytical ultracentrifugation and gel filtration analysis indicate the nucleotide exchange repressed SH3(E)-DH fragment is exclusively monomeric at micromolar concentrations, more than 100 times greater than used in the in vitro nucleotide exchange activity assay (figure 7). Consequently, it is unlikely that the DH-domain homodimer seen in our structure is physiologically relevant.

### DH – SH3(E) domain interface (closed interface)

**Figure 8:**
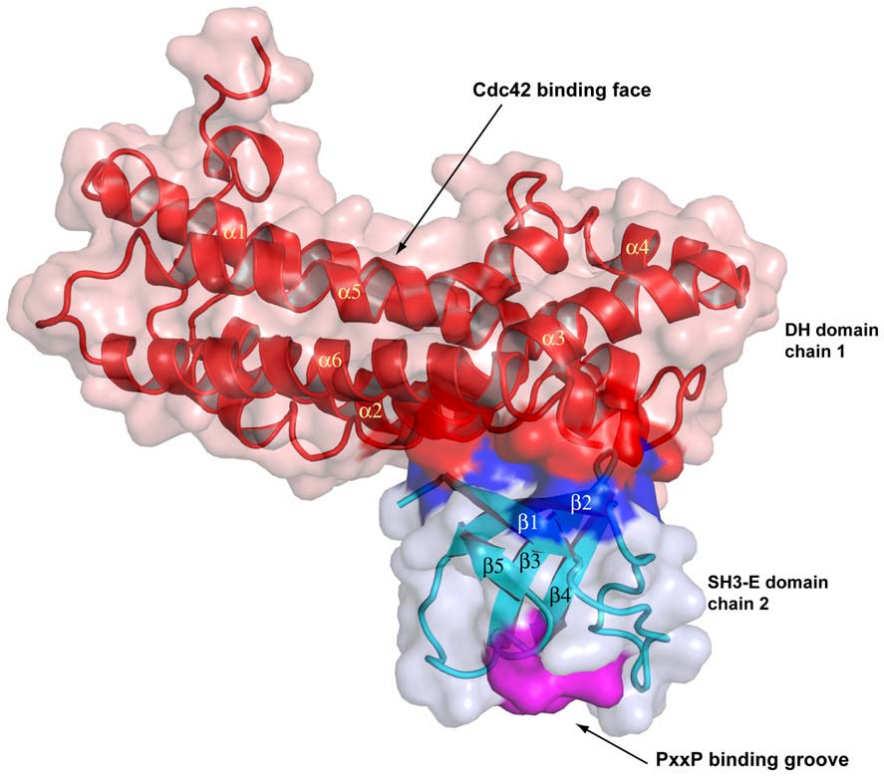
Surface representation of intersectin-1L DH domain – SH3(E) domain interaction. The DH domain from one monomer is in red, and the SH3(E) domain from the other monomer is in blue. The SH3(E) domain binds to the DH domain on the side of the molecule opposite the Cdc42 binding site (labeled). The polyproline type II helix binding groove on the SH3E domain (purple) does not overlap with the DH domain- SH3(E) domain interface.

The SH3(E) domain from one monomer forms an intermolecular interaction with the C-terminal half of the DH domain from the second monomer. This interaction occurs side of the DH-domain opposite the Cdc42 GTPase binding site. It buries 534 Å^2^ of surface area on the DH domain, and involves contacts with the first half of α2, the middle fragment of α3, and one face of the c-terminal region of α6 (with the other face of α6 interacting with the DH domain from the other chain). The first and second β-strands, and the small loop between the third and fourth β-strands of the SH3(E) domain are involved in the interactions with the DH domain (figure 8). Specifically, in β1 of SH3(E) Gln-1152 interacts with three residues of α6 of the DH domain, forming a hydrogen bond with Glu-1419 as well as contacting Lys-1420 and Ser-1423. In addition, Ile-1154 of β1 contacts Leu-1426 in α6 of the DH domain. In β2 of SH3(E), both Gln-1173 and Ile-1174 hydrogen bond to Glu-1427 of α6 of the DH domain. The highly conserved Ile-1174 contacts several other residues in α6 of the DH domain, including Lys-1420, Ser-1423, Asp-1424, and Leu-1426. Other residues in β2 of SH3(E) making contacts with the DH domain include Asn 1176, which interacts with Lys-1420 and Cys 1328 (α3) and Leu-1178 which interacts with Arg-1324 (α3). Residues from the small loop between the third and fourth β-strands of the SH3(E) domain that are involved in the interactions with the DH domain include Glu-1189, which interacts with Arg-1324, Cys-1328 (α3) and Met-1273 (α2), as well as Ser-1191, which interacts with two residues from α2 of the DH domain (Lys-1269 and Met-1273).

The intersectin-1L SH3(E)/DH domain interaction observed here is in contrast to that observed in the crystal structure of autoinhibited Asef, an SH3 domain containing GEF specific for Rac [29]. In the latter structure, the Asef SH3 domain was shown to form an intramolecular interaction with the DH domain in a manner that blocks the Rac-binding site. Furthermore, the RT-loop (the loop between β1 and β2) and the C-terminal portion of the Asef SH3 domain are involved in the interactions. As such, there is very little overlap between the DH domain binding surfaces of the SH3 domain from Asef and SH3(E) from intersectin-1L, suggesting that these GEFs utilize distinct mechanisms of autoinhibition.

Phe-1169, Trp-1185, and Tyr-1201 of the intersectin-1L SH3(E) domain correspond to three aromatic residues that form a series of ridges and grooves on the domain surface, against which a polyproline type II helix (PPII) containing a PxxP motif can pack [51]. The DH domain binding surface of SH3(E) does not overlap with the PxxP-binding groove (figure 8). Trp-1185 and Tyr-1201 are involved minor crystal contacts, while Phe-1169 is fully exposed to the solvent region.

### The hinge-loop

The probable “hinge-loop” region that would exist in a different conformation in the case of a monomeric SH3(E)-DH domain fragment must be long enough for the SH3(E) domain to fold back to the same peptide chain, a distance of approximately 50 Å. Although the linker between the SH3(E) domain and the DH domain is well ordered and forms multiple bridging contacts in crystallographic dimer, fully extended it could stretch up to 74 Å (assuming an average of 3.5 Å between α-carbons). Blockage or occlusion of the GTPase binding site by the extended linker in the SH3(E)-DH domain monomer could inhibit DH domain GEF activity.

## Discussion

The GEF activity of intersectin-1L is controlled by a core autoinhibitory interaction involving the fifth SH3 domain (SH3(E)) and the catalytic DH domain The crystal structure of the SH3(E)-DH domain fragment presented here reveals a homodimer in a domain swapped arrangement with the SH3 from one monomer forming an intermolecular interaction with the DH domain of the other monomer. While SH3-induced dimerization of the DH domain would obscure the Cdc42 binding interface, it is unlikely that this is the mechanism by which intersectin-1L GEF activity is autoinhibited. Both analytical ultracentrifugation and size-exclusion chromatography indicate that the SH3(E)-DH fragment is fully monomeric at micromolar concentrations. Therefore, we propose that the actual autoinhibited structure involves the related intramolecular interaction between SH3(E) and the DH domain. In the biologically relevant, full-length intersectin the proposed positioning of the SH3(E) domain on the DH domain may require a shift of the PH domain to prevent a steric clash, which is directly C-terminal to the DH domain. Although the SH3(E)-DH-PH fragment is repressed (figure 1b), we were unable to crystallize this protein.

The effect on GEF activity of mutating the DH binding site in the SH3(E) domain would shed more light on the biological relevance of this observed interaction. Candidate residues for mutation in the SH3(E) domain should contribute at least 5% to the buried surface area upon complex formation and should not be conserved among intersectin SH3 domains (figure 2a). Such residues can be mutated to the corresponding residue in SH3(D), an SH3 domain that does not repress the DH domain when attached in cis. A prime candidate is Gln-1151 in β1 of SH3(E). Gln-1152 interacts with three residues in α6 in the DH domain and contributes 15% of the total 538 Å^2^ of buried surface area upon complex formation. Candidates in β2 include Asn-1776 and Leu-1778, which contribute 7% and 8% to the total buried surface area upon complex formation respectively, and contact residues in α3 and α6 in the DH domain. Finally Ser-1191 (between the third and fourth β-strands) contacts a couple of residues in α2 of the DH domain and contributes 12% of the total buried surface area upon complex formation. The effect on DH domain GEF activity of altering the length of the linker between the SH3(E) domain and the DH domain can also be investigated.

Generally, autoinhibition can be either “direct” or “indirect” [54]. One or both of these mechanisms could explain the autoinhibition observed in intersectin-1L. The repressed state structures of Rho-GEFs Vav1 and Asef are prototypical examples of direct autoinhibition [13] [29]. For both of these cases, an N-terminal region (an Acidic domain) containing a tyrosine for Vav1, and an SH3 domain for Asef) forms an intramolecular interaction with the catalytic DH domain and directly binds the GTPase binding site. Intersectin-1L appears to be autoinhibited by a different mechanism. In the structure described here, the SH3(E) domain-binding surface on the DH domain is at the c-terminal end and on the opposite side of the molecule relative to the GTPase binding site. Thus, the SH3 domain and the GTPase do not compete for the same binding site on the DH domain. In the proposed monomeric structure of the SH3(E) – DH domain fragment, the 21 amino acid “hinge-loop” would exist in a conformation allowing the SH3(E) domain to fold back to the same peptide chain, a distance of approximately 50 Å. The implications of this conformational shift in the linker are two-fold. Firstly, conformational strain may be placed on the catalytic DH domain, which could lead to structural perturbations in the active site, locking it in a repressed conformation that is unable to bind Cdc42. This mechanism is similar to that observed in the repressed state Src kinase, in which intramolecular interactions lock the kinase into an inactive conformation, rather than block the active site [55]. However, a comparison of the crystallographically unique DH domain fragments reported here with the DH domain of human intersectin-1L complexed with Cdc42 result in an average pairwise RMSD value of less than 1.0 Å. Unless exceptional local rearrangements of key residues at the DH domain – Cdc42 interface occur, our observations do not support a model in which binding of SH3(E) to the intersectin DH domain alters the conformation of the DH domain. It is more likely that the linker crosses-over the Cdc42 binding face on the DH domain, sterically hindering Cdc42 binding. Disruption of the SH3(E) – DH domain intramolecular interaction (possibly by post-translational modification, or binding of a competitive activating ligand) would permit Cdc42 binding and subsequent nucleotide exchange.

Several synthetic guanine nucleotide exchange factors utilizing the DH domain of intersectin-1L have been engineered using some of the basic principles of autoinhibition described above [56]. For example, a construct in which the DH domain was flanked at the N-terminus by the syntrophin PDZ domain and at the C-terminus by the syntrophin PDZ ligand (i.e., PDZdomain - 3 amino acid linker - DH domain - 3 amino acid linker –RRRESIV-COOH) was repressed 5 fold relative to the constitutively active DH fragment or mutants missing either the PDZ domain or PDZ-ligand. Similar to the SH3(E)-DH intramolecular interaction observed in our structure, the PDZ domain does not compete with Cdc42 for the same binding site on the catalytic DH domain. Rather, the results indicate that the intramolecular PDZ-ligand interaction sterically occludes or conformationally disrupts the DH domain. Accordingly, addition of PDZ ligand in trans relieved repression, increasing Cdc42 exchange activity.

Our crystal structure indicates that the DH domain binding surface in SH3(E) does not overlap with the proline-peptide (PxxP) binding groove. This observation is consistent with data showing mutations in the PxxP binding groove in SH3(E) that disrupt SH3-PxxP binding have no affect on inhibition of exchange activity *in vitro* [47]. Thus, the SH3(E) domain has two binding surfaces with distinct functions. One surface docks against the catalytic DH domain to regulate GEF activity and the another binds proteins containing PPII helices, perhaps for cellular targeting. As mentioned previously, the SH3 domains of intersectin have been shown to target dynamin to endocytic complexes. Furthermore, binding of dynamin to the intersectin SH3 domains has no effect on the inhibitory activity of the SH3 domains in an *in vitro* nucleotide exchange activity [47]. Although the proline-rich region of N-WASP has been shown to activate immunoprecipitated intersectin-1L [43], this activation is not seen with recombinant intersectin-1L fragments [47]. It has been suggested that binding of an additional, not yet identified protein may be necessary to recapitulate the observed activation in a fully pure system [47]. Another possibility is amino terminal components of intersectin-1L not present in our recombinant fragments could play a role in the regulation of exchange activity. Other activators of intersectin-1L have been identified (i.e., EphB2, Numb), but the interacting domains have yet to be mapped [55], [56].

Although we have identified the core, minimal inhibitory fragment, it is likely that inhibition of the biologically relevant, full-length intersectin is further modulated by its other domains. Similar to the intersectins, Vav proteins are multi-domain GEFs. The GEF activity of the Vav1 DH domain is controlled by two coupled processes. Firstly, the DH active site is directly, but weakly, inhibited through an interaction from the N-terminally adjacent Acidic domain. Secondly, this core interaction is strengthened 10-fold by the contacts of the calponin homology (CH) domain with the Acidic, PH, and DH domains [32]. Studies of other multidomain proteins have shown that the structure of Vav1, with a core autoinhibitory fragment whose activity is modulated by other domain interactions, is widespread [42] [57] [58]. Our work on intersectin-1L is far from a complete description of its inhibition, but rather a start of what is likely to be the base element in a more complicated regulated network of interactions. The GEF activity of the DH domain of intersectin-1L in the context of the full-length protein (i.e., outside of the SH3 domain/DH-PH domain cassette) has yet to be determined. Of particular interest will be the effect of the coiled-coiled domain. It has been demonstrated that this domain mediates intersectin oliogomerization [44], a mechanism that has been shown to regulate the activity of other exchange factors of the Dbl family [59].

## Materials and Methods

### Crystallization and Structure Determination of Intersectin SH3(E)-DH (1151-1431)

Crystals were grown at room temperature in hanging drops by mixing 2 µl of 10 mg/ml protein with 2 µl of reservoir buffer (1.7 M LiSO_4_, 0.1 M Hepes pH 7.8). Hexagonal-shaped crystals routinely appeared after 72 hours of equilibration. Crystals were cryoprotected in precipitating reservoir solution enriched in 10% glycerol before flash-freezing to 100 K. A dataset was collected at the Advanced Light Source (ALS) at beamline 8.3.1, using an ADSC Q315r CCD detector. The data was processed with the HKL 2000 program suite [60]. The protein crystallized in space group P3_2_21 (a = b = 67.044 Å, α = β = 90°, γ = 120°) with 2 molecules per asymmetric unit. The structure was solved by molecular replacement with Phaser [61] using the Intersectin DH domain (1KI1) and SEM SH3 domain (1SEM) as search models. Manual model rebuilding was done with Coot [62], and structure refinement and the addition of waters was done with CNS [63]. The calculated electron densities for amino acids 1427 to 1431 of chain A, and 1150 to 1203, 1428 to1431 of chain B were not clear and consequently were omitted from the refinement process. 91.2% of residues are in the most favored region of the Ramachandran plot with 8.8% of residues in the additionally favored regions. Molecular graphics in the figures were generated with Pymol [64]. Protein interface and surface calculations were performed with EMBL-EBI PISA [65] [66].

### Protein Expression Plasmids

Intersectin fragments were cloned by polymerase chain reaction from a mouse intersectin 1L clone, kindly provided by JL Zamanian [47]. These fragments (SH3ABCDE-DHPH (741–1574), SH3CDE-DHPH (998–1574), SH3DE-DHPH (1070–1574), SH3E-DHPH (1151–1574), SH3E-DH (1151–1431), SH3ABC-DHPH (741–1069, 1222–1574), SH3D-DHPH (1070–1150, 1222–1574), DHPH (1222–1574), DH (1222–1431)) were subcloned into a modified pET-19b bacterial expression vector (Novagen), encoding tobacco etch virus (TEV) protease cleavable N-terminal hexahistidine tag fusion proteins. The described mouse intersectin-1L fragments have greater than 95% sequence identity to their human intersectin-1L counterpart.

### Protein Expression and Purification

#### GEFS

The pET-19(b) based constructs were used to transform *Escherichia coli* BL21(DE3)RIL cells. Transformants were grown at 37°C in Luria-Bertaini media (containing 100 mg/ml ampicillin) to an A_600_ of 0.6. IPTG was then added to the culture to a final concentration of 0.2 mM to induce protein expression. Growth was continued for an additional 4 hours, after which the cells were harvested by centrifugation. Cell pellets were lysed by sonication, and hexahistadine fusion proteins were purified by Ni-NTA affinity chromatography (Qiagen). Hexahistidine tags were removed by incubation with hexahistidine-tagged TEV protease at room temperature. Uncleaved protein, free hexahistidine, and hexahistidine-tagged TEV protease were removed by subsequent incubation with Ni-NTA resin. The GEFS were further purified by size-exclusion chromatography, with columns Superdex 200 or Superdex 75 (GE Biosciences) equilibrated in 150 mM NaCl, 20 mM Tris pH 8.3, and 1 mM TCEP.

#### Cdc42

A fragment of human Cdc42 (residues 1–179) was expressed as a hexahistidine fusion and purified as described above for GEFs, with the exception of the replacement of size-exclusion chromatography with anion-exchange chromatography on a Source Q column (Amersham). Residual bound nucleotide was removed by dialysis in 20 mM Tris, 50 mM NaCl, 5 mM EDTA, 2 mM DTT, pH 7.5. Cdc42 was preloaded with GDP by incubation with excess nucleotide. Nucleotide exchange was quenched by addition of 50-fold molar excess of MgCl_2_. Excess nucleotide was removed by dialysis into GEF Assay Buffer (20 mM Tris, 50 mM NaCl, 10 mM MgCl_2_, 1% glycerol, 1 mM DTT, pH 7.5).

#### *In vitro* nucleotide exchange assay

Relative activities of Intersectin GEF fragments were quantified with an assay that measures an increase in fluorescence observed following the incorporation of mant-GDP into Cdc42 [56]. Measurements were made with a SpectraMax Gemini XS (Molecular Devices) fluorescence multi-well plate reader (25°C, excitation: 360 nm, emission: 440 nm). Solutions were pre-equilibrated at 25°C for 10 minutes, and the reaction was initiated by mixing solutions of GEF/mant-GDP and GDP-loaded Cdc42. Final concentrations were 1 µM Cdc42(GDP), 25 nM GEF, 400 nM mant-GDP in GEF Assay Buffer. Activity was quantified by determining the slope of the initial linear phase of the exchange reaction, and normalized to reactions involving no GEF and DH, or DHPH alone [56].

#### Analytical Gel Filtration

100 µl of SH3(E)-DH protein at 10 mg/mL was loaded onto a Superdex-75 HR 10/30 column at 0.5 ml/minute. Elution volume of protein was monitored by absorbance at 280 nm. The column was equilibrated in 150 mM NaCl, 20 mM Tris pH 8.3, 1 mM TCEP, and had been calibrated with proteins from the GE Life Sciences LMW Gel Filtration Calibration Kit.

#### Analytical Ultracentrifugation

SH3(E)-DH (Intersectin 1151–1431) was monitored at 280 nm at sufficient concentration to give an absorbance reading of 0.3–0.6 (8 µM-17 µM). Samples were centrifuged at 14,000, 20,000, and 28,000 rpm (in succession) for 22 hours at each speed (1 scan/hour). These experiments were done at 20°C on a Beckman Optima XL-A ultracentrifuge with an An-60 Ti rotor. Data were processed using the program Reedit9 (Jeff Lary, National Analytical Ultracentrifuge Facility) and then fitted to effective reduced molecular weight (σ) values with WinNonlin [67]. Data and fitted curves were plotted and residuals calculated using MATLAB.
